# Colchicine effects on the ploidy level and morphological characters of Katokkon pepper (*Capsicum annuum* L.) from North Toraja, Indonesia

**DOI:** 10.1186/s43141-021-00131-4

**Published:** 2021-02-12

**Authors:** Reisky Megawati Tammu, Tri Rini Nuringtyas, Budi Setiadi Daryono

**Affiliations:** 1 Biology Education Department - Faculty of Education, Universitas Pelita Harapan, Jl. M. H. Thamrin, Lippo Karawaci, Tangerang, Banten 15811 Indonesia; 2 Faculty of Biology, Universitas Gadjah Mada, Jl. Teknika Selatan, Sekip Utara, Sleman, 55281 DI Yogyakarta, Indonesia

**Keywords:** *Capsicum annuum*, Colchicine, Fruit, Katokkon pepper, Mixoploid, Ploidy

## Abstract

**Background:**

Productivity and quality of crops can be increased through polyploidy plants induced by colchicine. The use of colchicine has never been applied to Katokkon pepper, a local red pepper variety from North Toraja Indonesia. This pepper is characterized by its unique shape like the small-bell pod type of paprika and its strong spicy taste. Therefore, this study aimed to determine the effects of colchicine treatment on the ploidy level and morphological characters of Katokkon pepper.

**Results:**

Flow cytometer analysis showed that all colchicine concentration treatments ranging from 0.025 to 0.1% with 24 h immersion time generated two plant groups based on their ploidy level; 50% of the total treatment plants was diploid while the rest plants were mixoploid. All colchicine treatment plants were significantly different in their width of stomata guard cells from the control plant. The effect of colchicine was also significantly on the plant height, thickness of fruit flesh, and the number of fruits per plant.

**Conclusions:**

The results of this study showed that colchicine treatment had a significant effect on the ploidy level and several morphological characters of the Katokkon pepper. The colchicine treatment increased the number of fruits per plant and thicker flesh of fruits but reduced the size and weight of Katokkon pepper. Our findings provide essential information to obtain tetraploid Katokkon plants through colchicine treatment in further research. This study benefits as a preliminary step for increasing the productivity and quality of the local red peppers in Indonesia.

## Background

Red pepper is a horticulture crop with various benefits in human life, including food, medicine, and the economy. It contains many essential nutrients and bioactive compounds with multiple benefits such as antioxidant, antimicrobial, antiviral, anti-inflammatory, and anticancer [11]. Nowadays, red pepper has become a daily consumption of Indonesian people within households and industries. The government sometimes has to import red pepper to meet domestic needs. In fact, in several areas of Indonesia, we can found many local red pepper varieties that have not been widely cultivated across the country. One of them is the Katokkon pepper or locally known as “Lada Katokkon,” which belongs to *Capsicum annuum* species and originated from North Toraja, Province of South Sulawesi, Indonesia [3]. *Capsicum annuum* has been known to have a 2x = 24 chromosome number [1]; however, detailed information about the karyotype of Katokkon pepper has not been reported so far. The Katokkon pepper has a spicy taste and unique shape similar to the “bell” pod type of paprika in a smaller size, and it is a part of larger-size pepper clusters in North Toraja which cultivated in an area of about 67 ha with 513 tons yield [30]. The improvement in pepper production still becomes one of the main performance indicators of the North Toraja Agriculture Department to increase people’s income and reduce the inflation rate [23]. Therefore, much effort is still needed in cultivation and other aspects to improve productivity and quality.

Advances in biological and agricultural sciences have supported crop productivity and quality through plant breeding. Plant breeding methods are very diverse, but the core is to select the best type among variants in terms of genotype and phenotype characters related to agronomic and economic value [2]. The goals of plant breeding for pepper are to improve the quality of fruits and plants include productivity, disease and pest resistance, pungency level, and nutritional content [8, 9].

Chromosome doubling can be induced by using colchicine, and it has been applied to various plants such as anise hyssop [31], banana [6], cassava [40], hot pepper [14], licorice [20], and potato [32]. The chromosome doubling that occurs in the plant cells is known as polyploidy. Polyploidy can improve the productivity and quality of the plant, such as higher yield and more tolerance to environmental stress [29]. In *Capsicum annuum*, it had been reported that the stomatal guard cell, chloroplast number, and root size were increased in the polyploid plants compared with the diploids [14]. The tetraploids resulted from colchicine treatment on *Platanus acerifolia* plants also have thicker and wider leaves [17]. In general, the tetraploid plants show an increase in organs and stomata size, trichome density, contents of secondary metabolites, and biomass properties [16, 27, 10].

The plant ploidy level can be determined quickly and accurately by using flow cytometry [34]. Moreover, stomata size is an anatomical character that can be easily used to identify the occurrence of polyploidy in plants [20]. The flow cytometry combined with stomata analysis can be used as an accurate ploidy level assessment with high-efficiency results. Thus, we conducted this study to determine the effect of colchicine in plant ploidy and morphological characters as preliminary data for the next project to increase the productivity and quality of Katokkon pepper. This pepper has high potential because of its strong spicy taste, unique shape, and rich in essential nutrients for health such as vitamin C, carotenoids, and capsaicin. Due to these potencies, Katokkon agriculture can be promoted to benefit the healthy food lifestyle and to increase the economic value of the society, especially for North Toraja farmers. Therefore, this study sought to find the best colchicine treatment condition to produce polyploid Katokkon plants with higher fruit quality.

## Methods

### Sample preparation and colchicine treatment

The Katokkon pepper seeds were obtained from the Agriculture and Fisheries Service of North Toraja Regency, Indonesia. This research was conducted using a completely randomized design, which consisted of three replicate of the control (K0) and the treatment with four variations of colchicine concentration, namely 0.025% (K1), 0.05% (K2), 0.075% (K3), and 0.1% (K4). A stock solution of 0.1% colchicine was prepared by dissolving 0.1 gram colchicine (BioChemica PanReac AppliChem, USA) into 100 mL distilled water. The stock solution was diluted to reach treatment concentration at 0.025%, 0.05%, and 0.075% according to the research design.

Subsequently, a total of 10 Katokkon pepper seeds were immersed in each 15 ml vials filled with corresponding colchicine solution treatments for 24 h. After the treatment, seeds were washed with distilled water and planted in the seedling tray containing soil medium. After 35 days, the seedlings were transferred into 10 × 10 cm polybags containing a mixture of soil and manure with a 2:1 ratio. The seedlings were watered three times a week. At 30 days after planting, the plants were transferred into the larger size polybags (35 × 35 cm) with the similar soil media. Plant maintenance was done by adding fertilization to 6- and 9-week-old plants. In this study, three replications of plants from each treatment sample were used for further data analysis.

### Stomata guard cell size analysis

The sample preparation of stomata guard cells was performed by clearing methods [28] and then observed under a light microscope connected with Optilab Advance (Miconos, Indonesia). Samples were obtained from the 1st, 2nd, and 3rd leaves (calculated from below) of the 1-month-old plant of controls and the treatments with an average length and width of 6.5 cm and 2.5 cm. The leaves were cut into small pieces (2 × 2 cm) and immersed with 70 % ethanol for 1 week to remove chlorophyll. At the end of the week, the ethanol solution was replaced with chloral hydrate. The vials containing all the components were heated for approximately 1 h until the leaves become transparent. The leaves were placed onto the object glass and observed under a light microscope. The length and width of the stomata guard cells were measured using the Optilab Viewer 2 with Image Raster software (Miconos, Indonesia). Three biological replications were applied for each treatment. All data obtained were analyzed using one-way ANOVA (*α* = 5%) and followed with the Duncan Multiple Range Test (DMRT) on the SPPS 22 Program.

### Ploidy level analysis using flow cytometer

The analysis of Katokkon ploidy level was carried out for 2-month-old plant using flow cytometer at Indonesian Institute of Sciences (LIPI) Laboratory. Samples were obtained from the 5th leaf (counted from below) of control and treatment plants with an average length and width of 13.3 cm and 4.8 cm. Each leaf sample was cut into 0.5 × 0.5 cm, placed into Petri dishes and treated with 250 μL CyStain Pi extracting buffer (Sysmex Partec, Germany). The leaves samples were chopped and smoothed using the razor blade. The extracts were filtered through sample tube filter to obtain 0.2 μL filtrate for each sample. A total of 800 μL propidium iodide dye was mixed into each sample. Subsequently, the tube was placed into the flow cytometer CyFlow® Space (Sysmex Partec, Germany) for analysis. Finally, the ploidy level was determined based on the peak pattern obtained [38].

### Morphological characters measurement and data analysis

The plant morphological characters measurement were carried out after the first harvest of Katokkon pepper, which was about 40 days after flowering (5-month-old plant). In the observation, the morphological characters were divided into two categories. First is the plant habitus of Katokkon pepper, including plant height, stem circumference, length, and width of the leaf. Second is the fruit characters of Katokkon pepper which included fruit length and diameter, fruit weight, flesh thickness, number of fruit per plant, number of seed per fruit, and the weight of 100 seeds. All data obtained were analyzed using one-way ANOVA (*α* = 5%) and followed by the Duncan Multiple Range Test (DMRT) on the SPPS 22 Program.

## Results

### Effect of colchicine on ploidy level of Katokkon pepper

In this study, we identified that all colchicine treatment plants were significantly different in their width of stomata guard cells from the control plants (Table 1). Based on the flow cytometer analysis, all colchicine treatments at a series of concentrations of 0.025 to 0.1% produced two types of ploidy levels, namely diploid and mixoploid (Table 2).

**Table 1:** The colchicine effect on length and width of stomata guard cells of Katokkon pepper

Colchicine concentration	Mean ± SD	
	Length (μm)	Width (μm)
K0 (0.000%)	31.48 ± 1.03^a^	9.08 ± 0.54^a^
K1 (0.025%)	32.27 ± 1.09^a^	9.60 ± 0.69^b^
K2 (0.050%)	31.78 ± 1.05^a^	9.53 ± 0.46^b^
K3 (0.075%)	32.17 ± 1.68^a^	9.47 ± 0.52^b^
K4 (0.100%)	33.51 ± 1.86^a^	9.60 ± 0.45^b^

Description: K0 (control); K1 (0.025%); K2 (0.05%); K3 (0.075%); K4 (0.1%). Data represent the mean ± SD of three replicates. The letters following the numbers within a column indicate a significantly different at *P* < 0.05 according to Duncan multiple range test. Plants were 1 month old

**Table 2:** The ploidy level of Katokkon plants based on flow cytometer analysis

No	Sample	**Cell count**	**Mean x**	**CV-x%**	Category
1.	K0	625	204.28	2.99	Diploid (2n)
2.	K1a	562	199.55	2.94	Diploid (2n)
3.	K1b	583	196.97	3.13	Mixoploid
		117	390.81	2.40	(2n,4n)
4.	K1c	565	200.93	2.81	Mixoploid
		101	401.41	2.00	(2n,4n)
5.	K2a	732	199.72	3.90	Diploid (2n)
6.	K2b	684	202.82	3.25	Mixoploid
		89	407.53	3.25	(2n,4n)
7.	K2c	736	196.30	4.70	Diploid (2n)
8.	K3a	614	195.15	3.96	Diploid (2n)
9.	K3b	611	196.68	4.19	Mixoploid
		144	395.79	2.81	(2n,4n)
10.	K3c	657	189.70	3.96	Diploid (2n)
11.	K4a	569	196.05	3.76	Mixoploid
		42	395.81	2.51	(2n,4n)
12.	K4b	746	192.99	4.72	Diploid (2n)
13.	K4c	688	202.19	4.01	Mixoploid
		105	409.42	2.71	(2n,4n)

Description: K0 (control); K1 (0.025%); K2 (0.05%); K3 (0.075%); K4 (0.1%). The letters a, b, and c following the K1 to K4 indicate the sample replicates for the treatment plant. Plants were 2 months old

### Effect of colchicine on morphological characters of the Katokkon pepper

The morphological characters of the Katokkon control and treatment plants were analyzed based on the plant habitus and fruit. The effect of colchicine in the plant characters was significantly different for the plant height only (Table 3). The fruit characters were significantly affected by colchicine except for the weight of 100 seeds (Table 4).

**Table 3:** The effects of colchicine on plant height, stem circumference, leaf length, and width of Katokkon pepper

Colchicine concentration	Mean ± SD			
	Plant height (cm)	Stem circumference (cm)	Leaf length (cm)	Leaf width (cm)
K0 (0.000%)	64.23 ± 4.52^a^	4.13 ± 0.15^a^	24.17 ± 1.89^a^	9.47 ± 1.10^a^
K1 (0.025%)	99.73 ± 4.97^c^	4.40 ± 0.17^a^	26.23 ± 1.66^a^	10.73 ± 1.01^a^
K2 (0.050%)	85.63 ± 3.44^b^	4.23 ± 0.25^a^	21.90 ± 2.97^a^	8.77 ± 1.46^a^
K3 (0.075%)	84.17 ± 4.80^b^	4.30 ± 0.26^a^	22.27 ± 2.93^a^	8.43 ± 0.67^a^
K4 (0.100%)	91.23 ± 4.79^b^	4.50 ± 0.10^a^	26.00 ± 2.18^a^	10.00 ± 0.70^a^

Description: K0 (Control); K1 (0.025%); K2 (0.05%); K3 (0.075%); K4 (0.1%). Data represent the mean ± SD of three replicates. The letters following the numbers within a column indicate a significantly different at *P* < 0.05 according to Duncan multiple range test. Plants were 5 months old

**Table 4:** The effect of colchicine on fruit characters of Katokkon pepper

CT	Measurement variables (mean ± SD)						
	FL (cm)	FD (cm)	FF (mm)	FW (g)	NF/P	NS/F	WS (g)
K0	3.30 ± 0.21^b^	3.05 ± 0.05^b^	1.70 ± 0.10^a^	6.49 ± 0.69^b^	16.33 ± 1.52^a^	33.33 ± 3.06^b^	0.32 ± 0.02^a^
K1	2.64 ± 0.24^a^	2.67 ± 0.20^a^	2.50 ± 0.30^b^	4.53 ± 0.17^a^	33.33 ± 1.52^b^	34.00 ± 3.00^b^	0.32 ± 0.03^a^
K2	2.78 ± 0.25^a^	2.55 ± 0.31^a^	2.37 ± 0.35^b^	4.35 ± 0.42^a^	46.33 ± 6.50^c^	36.00 ± 2.65^b^	0.33 ± 0.02^a^
K3	2.75 ± 0.31^a^	2.60 ± 0.07^a^	2.33 ± 0.15^b^	4.11 ± 0.90^a^	32.33 ± 4.04^b^	35.67 ± 4.04^b^	0.33 ± 0.03^a^
K4	2.87 ± 0.05^a^	2.62 ± 0.16^a^	2.32 ± 0.10^b^	4.08 ± 0.46^a^	46.67 ± 7.63^c^	27.33 ± 3.06^a^	0.33 ± 0.03^a^

Description: CT (colchicine treatment); K0 (control); K1 (0.025%); K2 (0.05%); K3 (0.075%); K4 (0.1%); FL (fruit length); FD (fruit diameter), FF (thickness of fruit flesh), FW (fruit weight); NF/P (number of fruit per plant); NS/F (number of seed per fruit); WS (weight of 100 seeds). Data represent the mean ± SD of three replicates. The letters following the numbers within a column indicate a significantly different at *P* < 0.05 according to Duncan multiple range test. Plants were 5 months old

## Discussion

### Effect of colchicine on ploidy level of Katokkon pepper

The colchicine treatment showed an effect on the ploidy level of Katokkon pepper by inducing the production of mixoploid plants. All colchicine concentration treatments ranging from 0.025 to 0.1% generated two plant groups based on their ploidy level; 50% of the total treatment plants was diploid (2n = 2x) while the rest plants were mixoploid (2n = 2x + 4x). Mixoploidy is a condition of the emergence of the cell with different chromosome ploidy in one organism [15]. The 0.025% (K1) and 0.1% (K4) colchicine concentration treatment produced more mixoploid plants than others. Our result is consistent with research reported that the colchicine treatment at 0.1% for 24 h on seeds of *Glycyrrhiza glabra* var. glandulifera produced the mixoploid plantlets [20]. A treatment of 0.1 % colchicine concentration with 24 h immersion time of *Humulus lupulus* L. explants also produced more mixoploid plants than diploid and tetraploid, with the percentage of 52.8%, 16.7%, and 19.4%, respectively [33]. The research reported that the optimal treatment to induce polyploids in *Rhododendron fortunei* was 0.1% colchicine concentration for 24 h based on its mortality rate and polyploid induction rate [19]. In the pummelo plants, the 0.1% concentration of colchicine again was observed to be the most efficient for tetraploid induction [7].

All mixoploid plants obtained in this study consisted of a mixture of two types of cells, namely diploid cell (2n) and tetraploid cell (4n). Similar results were obtained on the *Cajanus cajan* (L.) Mill sp. mixoploid plant, which consists of a mixture of 2n and 4n, formed by colchicine induction with a concentration of 10 mg/L and 15 mg/L [34]. Colchicine treatment 0.05% for 4 h against *Trigonella feonum-graecum* L. also produced mixoploid plants consisted of more than two types of cells, namely 2n, 4n, 6n, and 8n [24].

**Fig. 1:**
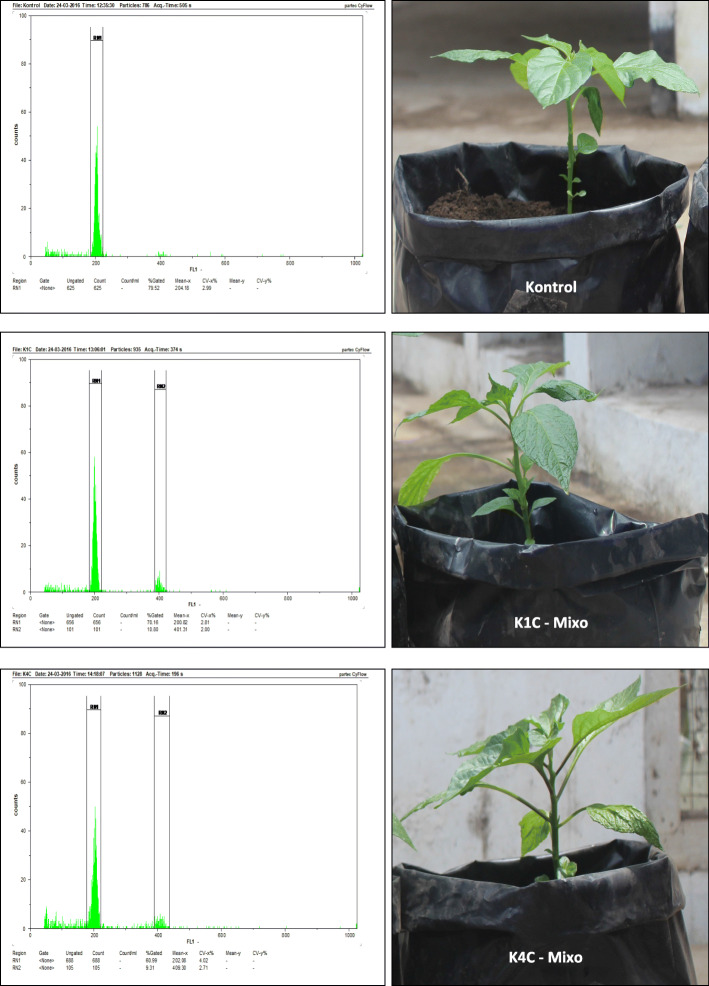
The ploidy level of Katokkon pepper plant based on flow cytometer. *Kontrol* (control diploid plant), *K1C Mixo* (0.025% colchicine treatment—mixoploid), and *K4C Mixo* (0.1% colchicine treatment—mixoploid)

The differences between flow cytometer results of diploid and mixoploid plants can be observed from the peaks of a histogram. The diploid plant (control) only had one peak detected on channel 200 while the mixoploid plant (K1C and K4C) had more than one peak, i.e., on channel 200 and 400 at the horizontal axis (Fig. 1). The histogram of the mixoploid plant showed that the peak value at channel 200 was higher than the peak value on channel 400. It means that the number of diploid cells (2n) is higher than tetraploid cells (4n) in the mixoploid plant. Similar results were found in *Brassica napus* mixoploid plants that the diploid cell is more dominant than the other cell types [15]. The mixoploid plants can be classified based on the relative number of diploid and tetraploid cells into grade 1, grade 2, and grade 3. Grade 1 is mixoploid plants with higher diploid cell number than tetraploid, grade 2 is for the plants with the same amount of diploid and tetraploid cells, and grade 3 is for plants with diploid cell number is less than tetraploid [13]. Based on these groupings, all of Katokkon mixoploid plants obtained in this study were mixoploid grade 1.

In this study, the Katokkon tetraploid plant (4n) has not been successfully produced. Instead, it achieved about 50% mixoploid plants out of total sample treatments. The emergence of mixoploid or chimeras instead of stable tetraploid plants shows that all cell layers of meristem tissues are not exposed by colchicine at the same time [18]. It may be influenced by the imprecise colchicine concentration or insufficient time immersion during the treatment process toward Katokkon seeds. In the chromosome doubling induction, the concentration and exposure time are important parameters to be optimized because colchicine binds poorly to plant tubulins [5]. In addition, the research found that chromosome doubling induction seemed highly affected and associated with the genotype of the plant [12, 21]. The exposure time of colchicine also needs to adjust with the mitosis period of cells within the actively growing tissue to produce a higher probability of polyploid plants [25]. It is suggested that we also consider the plant genotype and its mitosis period, in addition to the duration and concentration of colchicine to induce polyploidy successfully.

Based on the data of stomata guard cells size in Table 1, there was no significant difference between control and colchicine treatment in their lengths. However, the average of their widths showed a significant difference. The result indicates that colchicine treatment can cause a certain effect on the stomata guard cell size of the Katokkon plant. The observation of stomata length and density are suitable, quick, and easy methods of determining the ploidy level of many plants. The research showed a positive relation between ploidy level and stomatal guard cell length and width in *Citrus clementine* plant [26]. It also informed that the stomata size of the tetraploids larger but less than diploid plants [4, 21]. In comparison with diploid plants, the tetraploids showed an increase not only in the stomatal size but also the chloroplast numbers in guard cell [39].

### Effect of colchicine on morphological characters of Katokkon pepper

The plant height of colchicine treatment was significantly different from the control. The 0.025% colchicine concentration has a more significant effect on plant height than other concentrations. The stem circumference, leaf length, and width were not significantly different even though they showed small differences in mean values. This result showed that the variation of the colchicine concentration could have different effects on every plant character of Katokkon pepper, whether it increases or decreases its quality. The colchicine treatment was reported to show an increase in the plant height of *Pennisetum purpureum* [10]*.* It also was found on *Vicia cracca* that colchicine with several variations of concentration could cause an increase in plant height and performance [22]. On the contrary, colchicine reduced the plant height of *Impatiens balsamina* at three weeks after planting while plant stem diameter increased [35]. The tetraploid plants achieved by colchicine treatment showed an increase in phenotypic features such as leaf length and width, petiole, flower, and pollen diameters, but decreased in the pollen fertility [39].

**Fig. 2:**
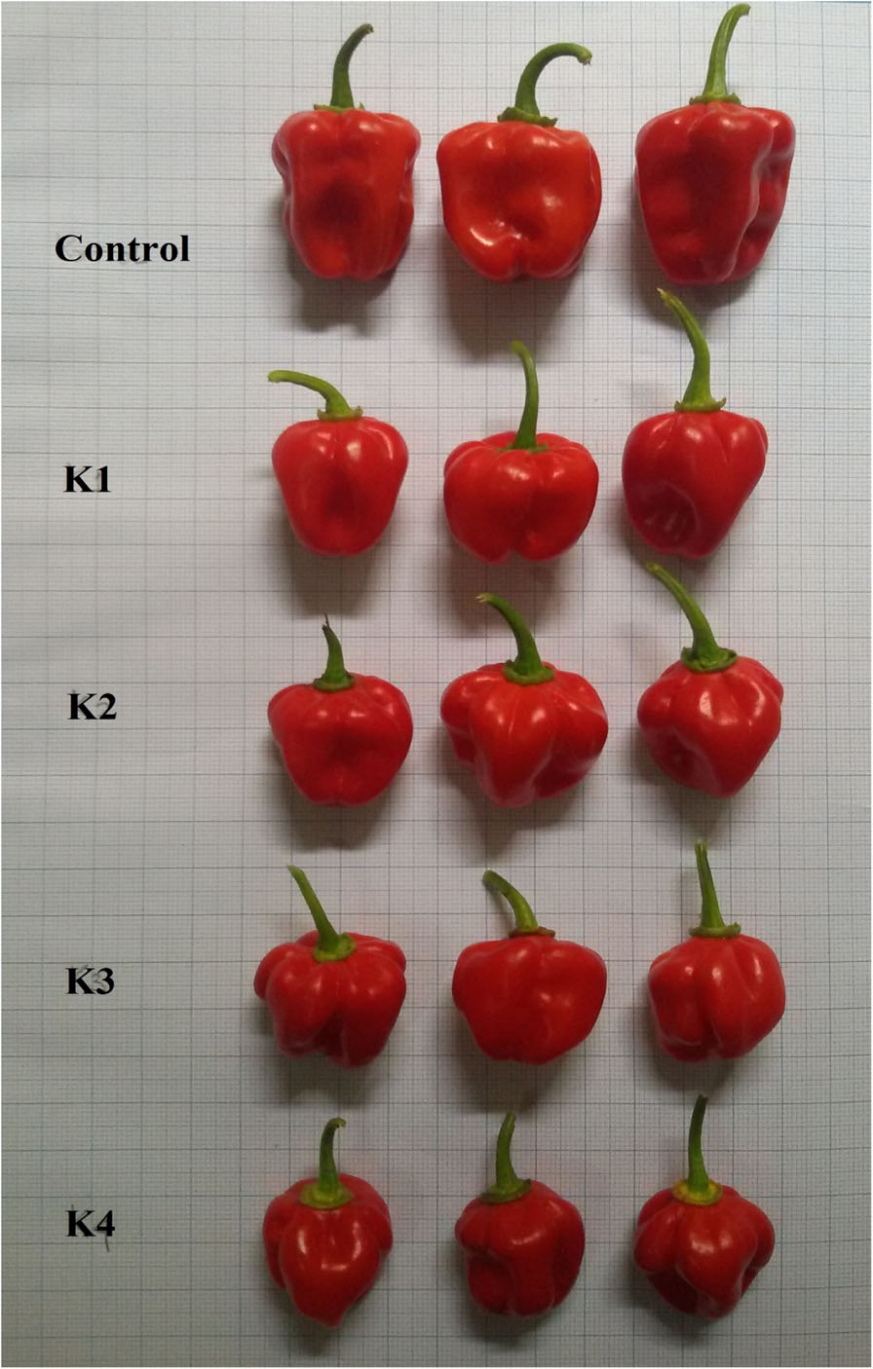
The Katokon fruits from control and colchicine concentration treatment plants. Description: control (K0); K1 (0.025%); K2 (0.05%); K3 (0.075%); and K4 (0.1%)

Observation of the fruit showed that the length and diameter of the control were higher than the results of the colchicine treatment (Fig. 2). However, the fruit thickness resulted from the treatment of colchicine concentration was higher and significantly different from the control (Table 4). The 0.025% colchicine concentration again has the most significant effect on the thickness of fruit flesh than other concentrations. Research also reported that the average thickness of kiwi fruit (*Actinidia chinensis*) resulted in colchicine induction were higher than controls [36]. The fruit weight of the control plants also was higher than the colchicine treatment. However, the plants treated by colchicine produce an average number of fruits of about two to three times higher than control plants. The research reported that colchicine increased fruit number while average fruit weight tended to decrease, but it was still significantly higher than controls [37]. The colchicine treatment with a concentration of 0.1% resulted in the smallest number of seeds per fruit. Although, the weight of 100 fruit seeds from colchicine treatment plants was not significantly different from the control.

Overall, the colchicine treatment can cause higher fruit numbers and thicker flesh but reduce their size and weight. In this study, it can be concluded that colchicine had significant effects on some of the morphological characters of Katokkon pepper, including the plant height, the thickness of fruit flesh, and the number of fruits per plant.

## Conclusions

In this study, we concluded that colchicine treatment had a significant effect on the ploidy level of the Katokkon pepper. All colchicine concentrations ranging from 0.025 to 0.1% with 24 h immersion time produced mixoploid plants. The colchicine treatment also can cause a certain effect on the stomata guard cell size of the Katokkon plant. Colchicine treatment affected the plant height, thickness of fruit flesh, and the number of fruits per plant. The number of fruits per plant and their flesh thickness increased but reduced their size and weight. Our findings provide essential information to obtain tetraploid Katokkon plants through colchicine treatment in further research. This study benefits as a preliminary step for increasing the productivity and quality of the local red peppers in Indonesia.

## Data Availability

Not applicable.
